# p53 protein, EGF receptor, and anti-p53 antibodies in serum from patients with occupationally derived lung cancer

**DOI:** 10.1038/sj.bjc.6690632

**Published:** 1999-08

**Authors:** J Schneider, P Presek, A Braun, P Bauer, N Konietzko, B Wiesner, H-J Woitowitz

**Affiliations:** Institut und Poliklinik für Arbeits- und Sozialmedizin, Justus-Liebig Universität Giessen, Aulweg 129/III, Giessen, 35385, Germany; Rudolf-Buchheim-Institut für Pharmakologie, Justus-Liebig Universität Giessen, Frankfurter Str. 107, Giessen, 35392, Germany; Ruhrlandklinik, Pneumologie, Universitätsklinik Essen, Tüschener Weg 40, Essen-Heidhausen, 45239, Germany; Klinik für Lungenkrankheiten und Tuberkulose, Robert-Koch-Allee 9, Bad Berka, 99437, Germany

**Keywords:** p53-protein, EGF-R, anti-p53 antibodies, serum, occupational, lung cancer

## Abstract

The oncogene product epidermal growth factor receptor (EGF-R), the tumour suppressor gene product p53 and anti-p53 antibodies are detectable in the serum of certain cancer patients. Increased levels of some of these products were reported in lung cancer patients after occupational asbestos exposure and after exposure to polycyclic aromatic hydrocarbons or vinylchloride. In the first step, this study investigated the possible diagnostic value of serum EGF-R, p53-protein and anti-p53 antibodies, measured by an enzyme-linked immunosorbent assay, in lung tumour patients. In addition to being investigated on a molecular epidemiological basis, these parameters were examined as biomarkers of carcinogenesis, especially with regard to asbestos incorporation effects or of radon-induced lung cancers. Also, a possible effect of cigarette smoking and age dependence were studied. A total of 116 male patients with lung or pleural tumours were examined. The histological classification was four small-cell cancers, six large-cell cancers, 32 adenocarcinomas, 47 squamous carcinomas, 12 mixed lung carcinomas, five diffuse malignant mesotheliomas and ten lung metastasis of extrapulmonary tumours. Twenty-two lung cancers and all mesotheliomas were related to asbestos, 22 lung cancers were related to ionizing radiation and 61 patients had cigarette smoke-related lung cancer. Besides these patients 50 male patients with non-malignant lung or pleural diseases were included; of the latter eight subjects suffered from asbestosis. Controls were 129 male subjects without any lung disease. No significantly elevated or decreased serum values for p53 protein, EGF-R, or anti-p53 antibodies as a function of histological tumour type, age, or degree and type of exposure (asbestos, smoking, ionizing radiation) could be found. The utility of p53-protein, EGF-R and anti-p53 antibodies as routine biomarkers for screening occupationally derived lung cancers is limited. © 1999 Cancer Research Campaign


*British Journal of Cancer* (1999) **80**(12), 1987–1994
© 1999 Cancer Research Campaign
Article no. bjoc.1999.0632

### p53 protein, EGF receptor, and anti-p53 antibodies in serum from patients with occupationally derived lung cancer

**J Schneider[1], P Presek[2], A Braun[2], P Bauer[3], N Konietzko[3], B Wiesner[4] and H-J Woitowitz[1]**

[1]Institut und Poliklinik für Arbeits- und Sozialmedizin, Justus-Liebig Universität Giessen, Aulweg 129/III, 35385 Giessen, Germany; [2]Rudolf-Buchheim-Institut für Pharmakologie, Justus-Liebig Universität Giessen, Frankfurter Str. 107, 35392 Giessen, Germany; [3]Ruhrlandklinik, Pneumologie, Universitätsklinik Essen, Tüschener Weg 40, 45239 Essen-Heidhausen, Germany; [4]Klinik für Lungenkrankheiten und Tuberkulose, Robert-Koch-Allee 9, 99437 Bad Berka, Germany

**Summary** The oncogene product epidermal growth factor receptor (EGF-R), the tumour suppressor gene product p53 and anti-p53 antibodies are detectable in the serum of certain cancer patients. Increased levels of some of these products were reported in lung cancer patients after occupational asbestos exposure and after exposure to polycyclic aromatic hydrocarbons or vinylchloride. In the first step, this study investigated the possible diagnostic value of serum EGF-R, p53-protein and anti-p53 antibodies, measured by an enzyme-linked immunosorbent assay, in lung tumour patients. In addition to being investigated on a molecular epidemiological basis, these parameters were examined as biomarkers of carcinogenesis, especially with regard to asbestos incorporation effects or of radon-induced lung cancers. Also, a possible effect of cigarette smoking and age dependence were studied. A total of 116 male patients with lung or pleural tumours were examined. The histological classification was four small-cell cancers, six large-cell cancers, 32 adenocarcinomas, 47 squamous carcinomas, 12 mixed lung carcinomas, five diffuse malignant mesotheliomas and ten lung metastasis of extrapulmonary tumours. Twenty-two lung cancers and all mesotheliomas were related to asbestos, 22 lung cancers were related to ionizing radiation and 61 patients had cigarette smoke-related lung cancer. Besides these patients 50 male patients with non-malignant lung or pleural diseases were included; of the latter eight subjects suffered from asbestosis. Controls were 129 male subjects without any lung disease. No significantly elevated or decreased serum values for p53 protein, EGF-R, or anti-p53 antibodies as a function of histological tumour type, age, or degree and type of exposure (asbestos, smoking, ionizing radiation) could be found. The utility of p53-protein, EGF-R and anti-p53 antibodies as routine biomarkers for screening occupationally derived lung cancers is limited.

**Keywords:** p53-protein; EGF-R; anti-p53 antibodies; serum; occupational; lung cancer

The number of compensated occupational cancers in Germany is still rising (Butz, 1996). The most frequent occupational cancers are lung carcinomas (45.9%) and mesotheliomas of the pleura, peritoneum and pericardium (37.5%). Currently, approximately two-thirds (68.6%) of all occupational cancers eligible for compensation are due to asbestos fibres. The second most common occupationally derived lung cancers (10.5%) are due to ionizing radiation, especially that caused by radon and its decay products. Aromatic amines account for 6.4% and polycyclic hydrocarbons 3.3% of cancer cases originating in the workplace (Butz, 1996). Because lung cancer occupies such a prominent place on the list of causes of cancer deaths, especially among men in the Western industrialized world, early detection of these tumours should be a major focus of interest. Improved secondary prevention by early detection may be a solution. To this end, special laboratory procedures employing bio-markers or bio-indicators are available.

Cancer is a result of a number of genetic alterations that disturb normal, controlled cell growth and differentiation (Harris, 1997). Proto-oncogenes and tumour suppressor genes and their products are key components of a cellular regulatory mechanism. The tumour suppressor gene product p53 and the epidermal growth factor receptor (EGF-R), an oncogene product, are associated with lung cancer pathogenesis and are likely to play a key role in the development of cancer (Harris and Hollstein, 1993). Since certain carcinogenic substances are known to cause particular mutations on certain genes (Vähäkangens et al, 1994), clues about the identity of these carcinogens should come from determination of oncogene and tumour suppressor gene products. In accordance with an elevated intracellular concentration, the proteins mentioned above have also been detected in the serum of tumour patients, some even before clinical manifestation of cancer (Brandt-Rauf et al, 1992; Luo et al, 1994). Autoimmune antibodies against p53 (anti-p53 AB) can also be detected in the serum of cancer patients (Harris and Hollstein, 1993). In light of the greater stability of antibodies as compared with the p53 protein product, the detection of anti-p53 AB might be a more efficient screening method.

In the present investigation, the suitability of EGF-R, p53 protein, or p53 autoimmune antibodies as bio-markers or bio-indicators detectable in serum was assessed as a method of early detection of malignant diseases of the lung induced by asbestos or radon and its products. Furthermore, the possible influence of non-occupational factors such as cigarette smoking was examined.

*Received 10 September 1998*
*Revised 12 January 1999*
*Accepted 16 February 1999*

*Correspondence to:* J Schneider

### PATIENTS AND METHODS

### Disease status and histological tumour type of the subjects

Within the framework of this multi-centre, molecular biology-based project, 129 male subjects (65.3 ± 5.4 years of age) without lung disease (Controls), 50 male patients (53.2 ± 13.2 years) with non-malignant lung/pleural disease (NMLD) and 116 male patients (63.3 ± 9.3 years) with lung or pleural tumours (Tumour-Patients) were examined. Among the patients with tumours were five with diffuse malignant pleuralmesothelioma (DMM) (61.6 ± 8.8 years) and ten with lung metastases of extrapulmonary primary tumours (55.0 ± 11.4 years). Histological classification of the lung carcinoma cases yielded four small-cell cancers (64.3 ± 7.6 years), six large-cell cancers (69.0 ± 9.0 years), 32 adenocarcinomas (62.7 ± 8.0 years), 47 squamous (64.8 ± 8.6) and 12 mixed or non-classifiable bronchial carcinomas (62.0 ± 12.7 years). Of the patients with NMLD, 26 had an acute inflammatory lung/pleural infection (49.4 ± 13.8 years) and 24 suffered from interstitial lung fibrosis (57.3 ± 11.7 years). The health status of each subject was confirmed in a clinical examination that included X-rays. All patients underwent bronchoscopy.

### Type of exposure (asbestos, ionizing radiation, polycyclic aromatic hydrocarbons)

To allow further discrimination between different kinds of exposure, several groups of tumour patients or patients suffering from fibrosis were created in a second evaluation step and compared to a non-exposed control group.

The tumour patients were as follows: 22 patients (64.5 ± 9.4 years) with asbestos-related lung cancer as defined in the list of occupational diseases (criteria for diagnosis: see DeVuyst, 1997), five patients (61.6 ± 6.8 years) with diffuse malignant mesotheliomas caused by asbestos dust, 61 patients (63.1 ± 9.0 years) with cigarette smoke-related lung cancer, i.e. lung cancer in cigarette smokers with no relevant exposure to other known occupational lung carcinogens and 21 former uranium miners of SDAG Wismut (67.5 ± 5.9 years) with lung cancer related to ionizing radiation (radon and its decay products) (criteria for diagnosis: see Eigenwillig, 1997).

The patients with fibrosis included eight subjects (64.5 ± 6.8 years) with asbestosis (criteria for diagnosis: see Parker, 1997) and 16 subjects (53.8 ± 12.1 years) suffering from lung interstitial fibrosis without any relevant asbestos exposure.

A non-exposed control group ($n$ = 13, 58.4 ± 7.0 years old) was defined as healthy subjects without exposure to carcinogenic (or fibrogenic) agents at the work place mentioned above, i.e. never having smoked or knowingly been exposed to asbestos dust or ionizing radiation.

### Age and smoking habits

Because of different ages and smoking habits in the various patient groups, a possible dependence of the p53 protein and EGF-R levels was examined. For the age comparison, the results for all serum samples ($n$ = 295) were evaluated. To examine the influence on smoking habits all sera of former and current smokers were analysed ($n$ = 238).

### Serum specimens

All sera were frozen following venipuncture, centrifuged and stored in liquid nitrogen until analysis. All analyses were performed in duplicate. p53 protein, anti-p53 antibodies and EGF-R were measured in serum by commercially available enzyme-linked immunosorbent assay (ELISA) kits (Oncogene Science, supplied by Dianova, Hamburg, Germany) according to the manufacturer’s instructions.

#### *p53*

The serum dilution was 1:5. The assay uses anti-p53 antibodies from rabbits as reporter antibodies, which react with mutant and wild-type human p53. The minimum concentration detectable was reportedly 10 pg $ml^{-1}$.

#### *Anti-p53 antibodies*

The serum dilution was 1:100. The assay was calibrated by three standards of various anti-p53 antibody concentrations. Samples were qualitatively judged positive or negative. A reaction was judged positive when the absorbance reading was equal to or greater than the absorbance of the low positive control value obtained with the ELISA kits.

#### *EGF-R*

The serum dilution was 1:4. The lower detection limit was 5 fmol $ml^{-1}$, as reported by the manufacturer. For all tests, absorbance was measured in each well by a spectrophotometric microplate reader (Bibbi Dunn, Asbach, Germany).

### Statistical analysis

Data were statistically analysed with SAS for Windows 6.08 (SAS, Cary, NC, USA). The Shapiro–Wilk test was used first to check for a normal distribution of values. Because of non-normal distribution in all groups, statistical comparisons between all groups were investigated by the non-parametric Wilcoxon test. A $P$-value of 0.05 or less was considered as statistically significant.

### RESULTS

### Comparative evaluation with respect to disease status and histological tumour type

The results of the measurement of p53 protein and EGF-R in serum showing minimum, maximum, median and mean values are given in Table 1. There were no significant differences in p53 protein or EGF-R concentrations in serum from patients with tumours as compared with patients having non-malignant lung disease or healthy control subjects.

In order to evaluate the suitability of these parameters as bio-markers for particular diagnostic types of malignant lung/pleural diseases, a possible influence of the histological tumour type on the expression of the bio-markers was examined. A summary of the values for p53 protein and EGF-R in serum as a function of histological tumour type are listed in Table 1. When analysed as a function of histological tumour type, there were no significant differences in serum values for both p53 protein and EGF-R, possibly owing to the small number of patients in some of the groups.

© 1999 Cancer Research Campaign

**Table 1** p53 and EGF-R concentrations in serum and detection of anti-p53 antibodies in healthy individuals and in patients with malignant or non-malignant lung disorders

| Subjects: histology of cancer | *n* | p53 (pg ml$^{-1}$) | | | | | EGF-R (fmol ml$^{-1}$) | | | | | Anti-p53 antibodies | | | |
|---|---|---|---|---|---|---|---|---|---|---|---|---|---|---|---|
| | | Min. | Max. | Med. | Mean | *P* | Min. | Max. | Med | Mean | *P* | Negative (*n*) | Positive (*n*) | Positive results (%) | *P* |
| Controls | 129 | 73 | 5153 | 469 | 579 | – | 18 | 67 | 36 | 37 | – | 114 | 15 | 12 | – |
| NMLD | 50 | 157 | 2127 | 514 | 560 | 0.85 | 21 | 156 | 39 | 41 | 0.19 | 44 | 6 | 12 | 0.85 |
| Tumour-Patients | 116 | 86 | 2628 | 541 | 578 | 0.25 | 18 | 138 | 37 | 39 | 0.25 | 94 | 22 | 19 | 0.15 |
| Small-cell LC | 4 | 86 | 1069 | 530 | 554 | 0.97 | 30 | 49 | 35 | 37 | 0.97 | 2 | 2 | 50 | 0.08 |
| Large-cell LC | 6 | 243 | 648 | 479 | 478 | 0.78 | 27 | 39 | 33 | 33 | 0.27 | 6 | 0 | 0 | 1.00 |
| Adenocarcinoma LC | 32 | 194 | 1531 | 521 | 600 | 0.28 | 18 | 56 | 37 | 38 | 0.41 | 26 | 6 | 19 | 0.38 |
| Squamous LC | 47 | 117 | 2628 | 574 | 611 | 0.13 | 20 | 69 | 38 | 38 | 0.43 | 36 | 11 | 23 | 0.09 |
| Mixed, non-classificable LC | 12 | 212 | 1120 | 408 | 550 | 0.93 | 26 | 138 | 36 | 44 | 0.73 | 10 | 2 | 17 | 0.64 |
| DMM | 5 | 400 | 684 | 508 | 528 | 0.65 | 28 | 58 | 36 | 41 | 0.49 | 5 | 0 | 0 | 1.00 |
| Lung-metastasis of extrapulmonary tumours | 10 | 201 | 772 | 455 | 461 | 0.40 | 24 | 60 | 40 | 42 | 0.11 | 9 | 1 | 10 | 1.00 |

DMM, diffuse malignant mesothelioma; NMLD, non-malignant lung diseases, i.e. fibrosis (*n* = 24) or inflammatory diseases (*n* = 26); LC, Lung cancer; Min, minimum; Max, maximum; Med, median.

**Table 2** p53 and EGF-R concentrations in serum and detectable anti-p53 antibodies as a function of various occupational or environmental factors

| Subjects and type of exposure | *n* | p53 (pg ml$^{-1}$) | | | | | EGF-R (fmol ml$^{-1}$) | | | | | Anti-p53 antibodies | | | |
|---|---|---|---|---|---|---|---|---|---|---|---|---|---|---|---|
| | | Min. | Max. | Med. | Mean | *P* | Min. | Max. | Med. | Mean | *P* | Negative (*n*) | Positive (*n*) | Positive results (%) | *P* |
| Healthy subjects | | | | | | | | | | | | | | | |
| Non-smokers without asbestos or ionizing radiation exposure | 13 | 231 | 874 | 527 | 541 | – | 25 | 44 | 35 | 35 | – | 11 | 2 | 15 | – |
| Tumour-patients | | | | | | | | | | | | | | | |
| Mesothelioma due to asbestos | 5 | 400 | 684 | 508 | 528 | 0.80 | 28 | 58 | 36 | 41 | 0.35 | 5 | 0 | 0 | 0.5 |
| LC due to asbestos | 22 | 117 | 2628 | 656 | 674 | 0.44 | 27 | 138 | 39 | 44 | 0.09 | 19 | 3 | 14 | 0.63 |
| LC due to ionizing radiation | 21 | 211 | 1326 | 564 | 632 | 0.48 | 23 | 50 | 37 | 37 | 0.47 | 18 | 3 | 14 | 0.65 |
| LC due to smoking | 61 | 86 | 1531 | 491 | 547 | 0.73 | 18 | 65 | 37 | 37 | 0.62 | 45 | 16 | 26 | 0.33 |
| Patients with lung fibrosis | | | | | | | | | | | | | | | |
| Asbestosis | 8 | 283 | 2127 | 476 | 666 | 0.71 | 25 | 52 | 35 | 37 | 0.66 | 7 | 1 | 13 | 0.68 |
| Lung fibrosis not due to asbestos | 16 | 293 | 1400 | 542 | 642 | 0.91 | 22 | 156 | 33 | 52 | 0.52 | 13 | 3 | 19 | 0.60 |

LC, lung cancer; Min, minimum; Max; maximum; Med, median.

The presence of antibodies against p53 protein (anti-p53 AB) was detected in 43 (= 14.6%) out of a total of 295 serum samples (Table 1). Antibodies against the p53 gene product were found in sera from half of the patients with small-cell carcinoma, which represents the highest prevalence. No anti-p53 AB was detected in sera from patients with large-cell bronchial carcinoma or diffuse malignant pleuramesothelioma. A number of serum samples from healthy subjects and patients with non-malignant lung disorders also contained anti-p53-AB (12% in each group). Taken together, these data showed no significant differences between the different groups.

### Comparative evaluation with respect to type of exposure

The central question of this study was the suitability of p53 protein, EGF-R and anti-p53 AB as markers for diseases due to asbestos fibres, ionizing radiation, or smoking. Table 2 shows minimum, maximum, median and mean values for both p53 protein and EGF-R, as well as the occurrence of anti-p53 AB and the results of biometrical tests for significance of data gathered from various patient groups, including non-smoking, healthy control subjects who had not been exposed to asbestos or ionizing radiation in the workplace. The tests applied showed no significant differences between all groups concerning the concentration of either p53 protein, EGF-R or the occurrence of anti-p53 antibodies in serum.

It must be taken into account that disorders arising from asbestos exposure cannot be differentiated clinically or pathologically from tumours, respectively, interstitial fibrosis originating from other causes. Our data also do not allow for this discrimination. In addition, lung cancer in former uranium miners caused by radon and its decay products had no effect on the analysed parameters.

### Age dependence of p53 protein and EGF-R concentrations in serum

Because of different ages in the various patient groups, a possible dependence on age for levels of p53 protein and EGF-R was

© 1999 Cancer Research Campaign

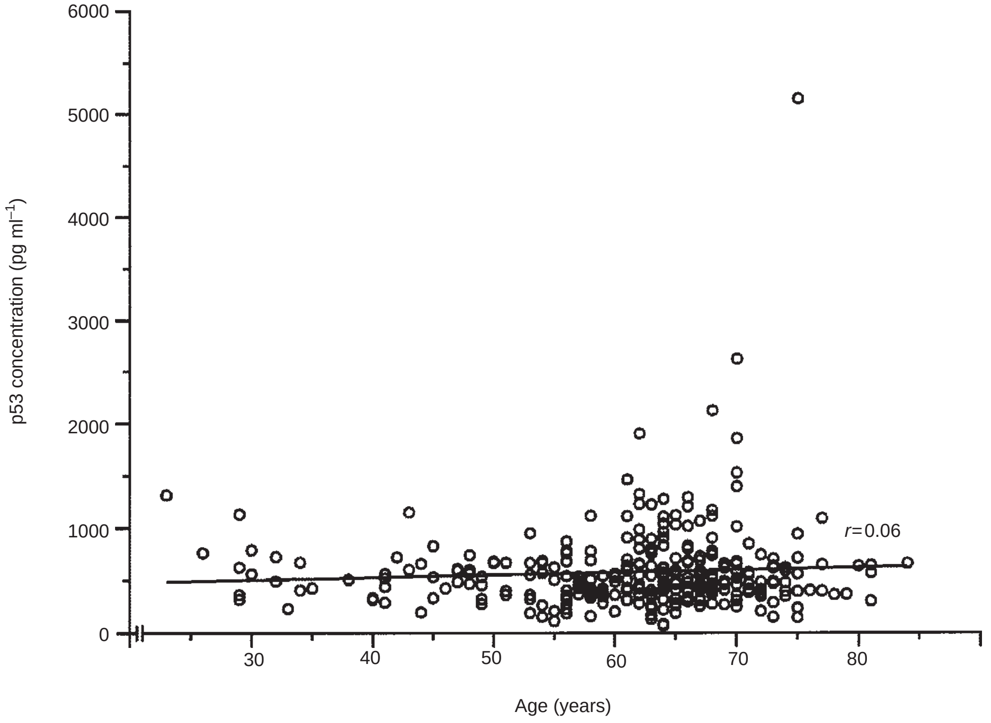

**Figure 1** p53 protein in serum as a function of age. p53 concentrations in sera of all paticipants ($n$ = 295) were measured as described in Methods. The linear regression is shown. The calculated correlation coefficient is $r$ = 0.06

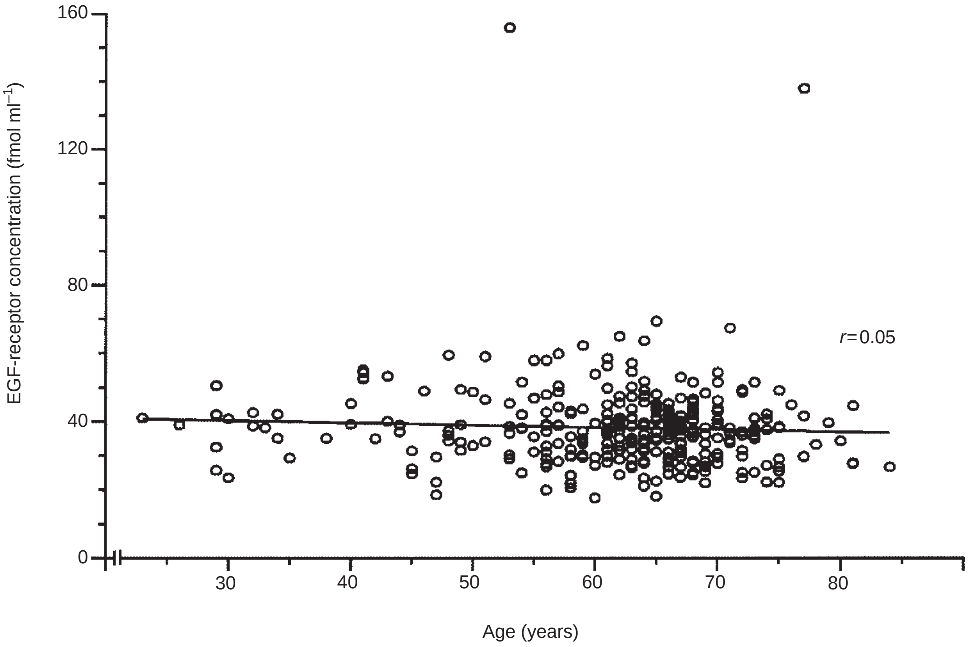

**Figure 2** EGF-R in serum as a function of age. EGF-R concentrations in sera of all participants ($n$ = 295) were measured as described in Methods. The linear regression is shown. The calculated correlation coefficient is $r$ = 0.05

examined. For this comparison, the results for all serum samples ($n$ = 295) were evaluated. There was no dependence on age for all three parameters tested. As can be seen in Figure 1 the highest value for p53 (5153 pg ml$^{-1}$) was obtained for a 75-year-old ex-smoker. Clinical examination of this patient revealed no obvious disorders. The highest concentrations of EGF-R in serum were measured for a non-smoking 53-year-old patient with idiopathic interstitial lung fibrosis (156 fmol ml$^{-1}$) and a 77-year-old patient (ex-smoker with 41 cumulative pack-years) with asbestos-induced lung cancer (138 fmol ml$^{-1}$) (Figure 2).

*© 1999 Cancer Research Campaign*

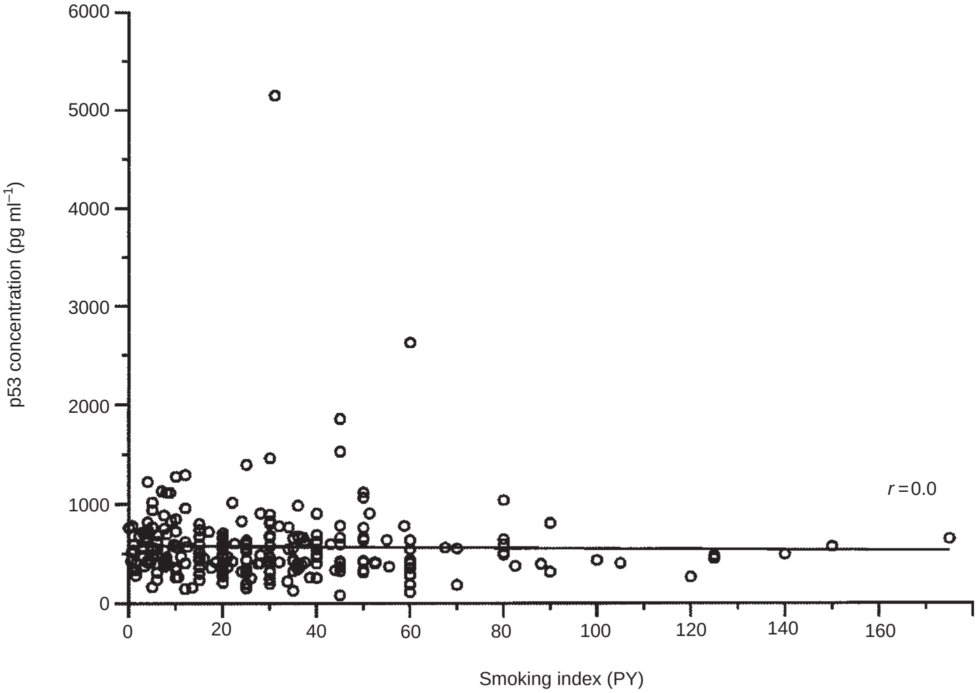

**Figure 3** p53 protein in serum as a function of cigarette consumption. p53 concentrations in sera of all individuals who had ever smoked ($n$ = 238) were measured as described in Methods (PY = pack years). The linear regression is shown. The calculated correlation coefficient is $r$ = 0.0

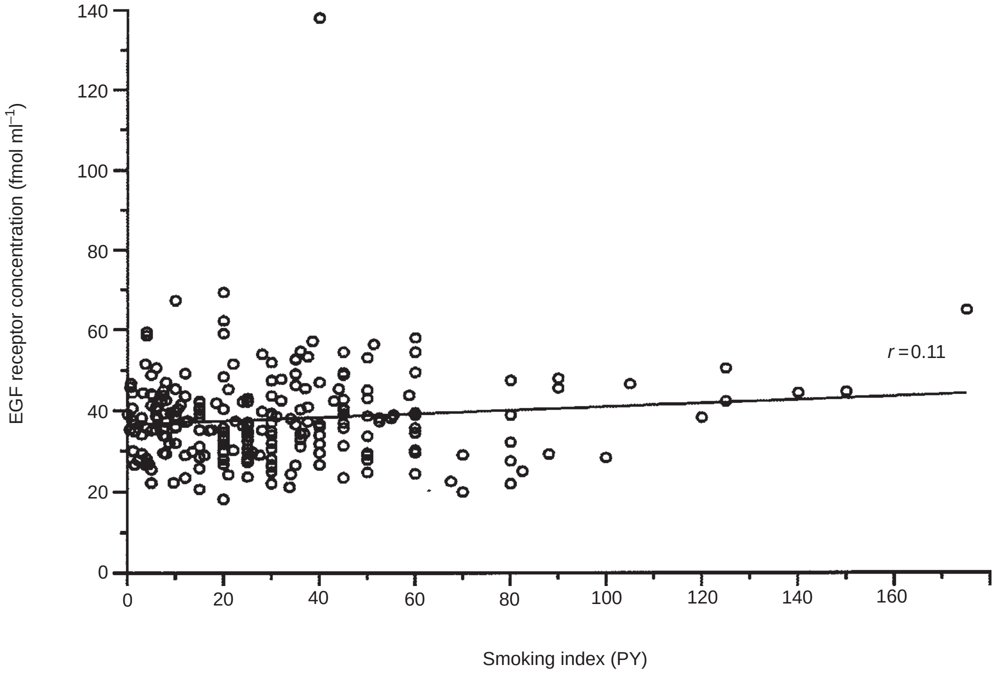

**Figure 4** EGF-R in serum as a function of cigarette consumption. EGF-R concentrations in sera of all individuals who had ever smoked ($n$ = 238) were measured as described in Methods (PY = pack years). The linear regression is shown. The calculated correlation coefficient is $r$ = 0.11

### Dependence of p53 protein and EGF-R concentration on smoking

The study population contained a total of 238 smokers or ex-smokers. To examine the dose–response relationship, the concentrations of p53 protein and EGF-R were plotted as a function of the cumulative time of exposure taken from patients' reported history of pack-years (PY). One PY is the equivalent of smoking about 20 cigarettes per day over the time period of 1 year. The results of this comparison are shown in Figure 3 and 4. There was no significant correlation between the cumulative dose of cigarette smoking (smoking index) and concentrations of the two

*© 1999 Cancer Research Campaign*

**Table 3** Anti-p53 antibody determination as a function of histological tumour type

| First author, year | Total LC | | Small-cell LC | | Non-small-cell LC Total | | Large-cell | | Adeno | | Squamous | | Mixed | |
|---|---|---|---|---|---|---|---|---|---|---|---|---|---|---|
| | *n* | % | *n* | % | *n* | % | *n* | % | *n* | % | *n* | % | *n* | % |
| Winter, 1992 | 6/46 | 13 | 4/40 | 10 | 2/6 | 33 | NA | – | NA | – | NA | – | NA | – |
| Winter, 1993 | 24/40 | 24 | 21/36 | 58 | 3/4 | 75 | NA | – | NA | – | NA | – | NA | – |
| Schlichtholz, 1994 | 10/42 | 24 | 4/9 | 44 | 6/33 | 18 | 2/5 | 40 | 2/10 | 20 | 2/18 | 11 | NA | – |
| Wild, 1995 | 16/136 | 12 | 1/7 | 14 | 15/129 | 12 | 0/8 | 0 | 3/49 | 6 | 11/70 | 16 | 1/2 | 50 |
| Trivers, 1995 | 2/15 | 13 | NA | – | NA | – | NA | – | NA | – | NA | – | NA | – |
| Rosenfeld, 1997 | 27/170 | 16 | 27/170 | 16 | NA | – | NA | – | NA | – | NA | – | NA | – |
| This study | 21/101 | 21 | 2/4 | 50 | 19/97 | 20 | 0/6 | 0 | 6/32 | 19 | 11/47 | 23 | 2/12 | 17 |

NA, not available; LC, Lung cancers.

bio-markers in serum. This is also true for anti-p53 antibodies (data not shown).

### DISCUSSION

Oncogene and tumour suppressor gene products have been linked to the occurrence of certain cancers (for overview see Barker and Vinson, 1990; Sugimura et al, 1991; Wynford-Thomas, 1991; Micelli et al, 1992; Burkhart, 1994; Lahdeaho et al, 1994; Trumper et al, 1994). The ubiquity and high frequency of p53 mutations in human malignancies is consistent with p53 protein being an important tumour suppressor gene product. Mutations in the p53 gene represent one of the most common genetic aberrations in lung cancer (Brambilla et al, 1993; Minna, 1993; Kaye et al, 1995). For example, mutations appear in 70–100% of small-cell carcinomas and in 40–60% of non-small-cell tumours (Minna, 1993; Kaye et al, 1995). The occurrence of p53 mutations has even been associated with a shortened patient survival (Horio et al, 1994), although this finding could not be verified by other authors (Van Zandwijk et al, 1995). Certain p53 mutations appeared to be specific for radon-associated lung cancer (Taylor et al, 1994). The protein product of the p53 gene represents an important tumour suppressor protein that is also detectable in serum (Fontanini et al, 1994). In the serum of 42% of lung cancer patients, both the wild-type and mutant forms of p53 were detected (Fontanini et al, 1994). Significantly higher concentrations of p53 protein were found in the serum of patients with advanced tumours.

A significant overexpression of p53 protein in sera has been reported in individuals after asbestos exposure than in people without such exposure (Nuorva et al, 1994). Lou and co-workers (1994) determined a higher mean concentration of p53 protein in sera of patients with asbestos-derived lung cancer as compared with sera from lung carcinoma patients who had no occupational asbestos exposure or with control sera from patients without cancer. This difference, however, did not prove to be significant. Husgafvel-Pursiainen et al (1997) mentioned that there was a statistically significant difference in the rate of p53 seropositivity between cancers following asbestos and silica exposure (36%) and the non-cancer controls (6%).

High levels of serum pantropic p53 proteins have also been associated with high risk for lung cancer in workers after a long duration of exposure to chromium compounds (Hanaoka et al, 1997). The authors suggested that this would be an early effect marker and a possible indicator of cancer risk.

Data in the literature concerning lung cancer and asbestos-associated diseases could not be corroborated in our investigation of sera from patients or exposed subjects (Tables 1 and 2). The measured values for p53 protein in serum from our patient groups are in the range of values reported in the literature (Fontanini et al, 1994; Luo et al, 1994), but there were no significant differences in serum levels between the groups we examined. There were also no differences as a function of histological tumour type, possibly owing to the scatter of the data.

No correlation was found between p53 protein concentration in serum and the occurrence of either asbestos-associated lung diseases or Schneeberg lung cancer (Table 2). Based on these results, the presence of p53 protein in serum does not appear to be a useful bio-marker for asbestos- or radon-induced lung cancer. Exposure to polycyclic aromatic hydrocarbons as a result of cigarette smoking also did not have an influence on the level of p53 protein in serum. The results of our investigation of p53 protein in serum based on the commercially available ELISA does not allow any aetiological differentiation of the origin of cancer.

In agreement with Segawa et al (1997), the levels of p53 protein in sera were not correlated with age and smoking index (Figures 1 and 3). The measurement of p53 protein serum levels was not found to be clinically useful neither for detection of small-cell lung cancer nor for detection of other histological types of lung cancer (Table 1).

Furthermore, Levesque et al (1996) could not confirm the presence of p53 protein in the serum of patients with lung cancer. In addition, these authors discussed possible analytical difficulties with the measurement of p53 protein in biological fluids. Thus, interference by heterophilic antibodies could nullify the results. They concluded that the detection of p53 protein in sera of lung cancer patients had to be interpreted with caution.

Besides the p53 protein, antibodies against the p53 protein have been detected in a large proportion of lung cancer patients (Harris and Hollstein, 1993; Winter et al, 1993; Angelopoulou et al, 1994; Mudenda et al, 1994; Preudhomme et al, 1994). The prevalence of anti-p53 antibodies in serum from lung cancer patients is reported between 15% (Wild et al, 1995) and about 30% (Schlichtholz et al, 1994; Lubin et al, 1995) and was also dependent on histological tumour type (Table 3).

 © 1999 Cancer Research Campaign

Antibodies to p53 protein have never been detected in the serum of healthy subjects (Rosenfeld et al, 1997). In some cases, anti-p53 antibodies were found in the serum of cancer patients before clinical manifestation of disease (Schlichtholz et al, 1994; Lubin et al, 1995; Trivers et al, 1995). Trivers and co-workers determined the presence of anti-p53 antibodies 4 months, in particular even 11.3 and 10.8 years, before diagnosis of cancer. Individuals with detectable anti-p53 antibodies had an improved survival compared with patients without these antibodies (Winter et al, 1993).

No significant differences were found between patients with malignant or benign lung disorders and healthy control subjects in our investigation of anti-p53 antibodies in serum (Table 1). There was also no dependence on histological tumour type. Also the type of exposure (asbestos, cigarette smoke, or radon and its decay products) also did not appear to have any influence on this parameter. There were no significant differences in the prevalence of anti-p53 antibodies between the various patient groups or between patients and healthy controls. It should be noted, however, that 15 (12%) healthy subjects and six (12%) patients with non-malignant disorders showed elevated levels of anti-p53 antibodies in serum. The predictive value of anti-p53 antibodies awaits the results of a planned follow-up.

Another oncogene product of interest is the EGF-R, which is the product of the erb B1 gene. An immunohistochemical overexpression is a common trait of human tumours, which has been observed for non-small-cell bronchial carcinomas (squamous cell carcinomas or adenocarcinomas) (Haeder et al, 1988; Sozi et al, 1991; Birrer and Brown, 1992; Pavelic et al, 1993; Rusch et al, 1993; Weissfeld et al, 1994; Brandt-Rauf et al, 1995; Sorscher et al, 1995). EGF-R could also be detected in serum. In our study, EGF-R levels were not elevated in patients with lung cancer or mesothelioma (Table 1). There were also no differences in EGF-R serum concentrations as a function of histological tumour type or age (Figure 2). Thus, the observations of Ambs et al (1989) concerning patients with kidney tumours or of Choi et al (1997) with stomach carcinoma patients could not be confirmed for lung cancer patients.

For causal analysis the reports from Partanen et al (1994*a*, 1994*b*) and Brandt-Rauf et al (1995) are important. Partanen and co-workers examined sera from 110 asbestosis patients for the appearance of EGF-R. Of these patients, 27 eventually developed bronchial carcinoma and three others pleuralmesothelioma. The EGF-R values in serum from these patients were significantly higher than those of control patients without asbestos exposure. Brandt-Rauf et al (1995) found elevated concentrations of EGF-R in seven out of 38 pneumokoniosis patients, among them three with lung carcinoma and one with mesothelioma.

A statistically significant overexpression of EGF-R in serum, however, could not be demonstrated in any group in our investigation. Based on our results, EGF-R cannot be viewed as a marker for early effects of occupational diseases due to asbestos or for radon-induced lung cancer.

### CONCLUSIONS

In the literature there is evidence supporting the utility of the tumour markers examined in this study with regards to the effects of certain carcinogenic substances found in the workplace. In contrast, our investigation found no significantly elevated or decreased serum values for p53 protein, EGF-R, or anti-p53 antibodies as a function of tumour type, histology, age, or degree and type of exposure (asbestos, smoking, ionizing radiation). Based on these results, routine use of the bio-markers examined as determinants of occupationally derived lung carcinomas cannot be recommended. Since anti-p53 antibodies were also found in the sera of subjects without clinical evidence of cancer, the predictive value of this potential bio-marker should only be evaluated after long-term follow-up.

### ACKNOWLEDGEMENTS

This work was supported by Hauptverband der gewerblichen Berufsgenossenschaften, Sankt Augustin, Germany.

### REFERENCES

Angelopoulou K, Diamandis KE, Sutherland DJ, Kellm JA and Bunting PS (1994) Prevalence of serum antibodies against the p53 tumor suppressor gene protein in various cancers. *Int J Cancer* **58**: 480–487

Ambs KE, Takahashi A, Hering F, Costa S and Huber PR (1989) Epidermal growth factor receptor in adenocarcinoma of the kidney. *Urol Res* **17**: 251–254

Barker S and Vinson GP (1990) Epidermal growth factor in breast cancer. *Int J Biochem* **22**: 939–945

Birrer MJ and Brown PH (1992) Application of molecular genetics to the early diagnosis and screening of lung cancer. *Cancer Res* **52**: 2658s–2664s

Brambilla E, Gazzeri S, Moro D, Caron de Fromental C, Gouyer V, Jacrot M and Brambilla C (1993) Immunhistochemical study of p53 in human lung carcinomas. *Am J Pathol* **143**: 199–210

Brandt-Rauf PW (1995) The c-erbB transmembrane growth factor receptors as serum biomarkers in human cancer studies. *Mutat Res* **333**: 203–208

Brandt-Rauf PW, Smith S, Hemminki K, Koskinen H, Vainio H, Niman H and Ford J (1992) Serum oncoproteins and growth factors in asbestosis and silicosis patients. *Int J Cancer* **50**: 881–885

Burkhart C (1994) Das Tumorsuppressorprotein p53. *Dtsch Ärzteblatt* **91**: 898–904

Butz M (1996) Beruflich verursachte Krebserkrankungen. Schriftenreihe des Hauptverbandes der gewerblichen Berufsgenossenschaften, St. Augustin, Germany

Choi JH, Oh JY, Ryu SK, Kim SJ, Lee NY, Kim KS, Yi SY, Shim KS and Han WS (1997) Detection of epidermal growth factor receptor in the serum of gastric carcinoma patients. *Cancer* **79**: 1879–1893

De Vuyst P (1997) Guidelines for attribution of lung cancer to asbestos. In: *Proceedings of an International Expert Meeting on Asbestos, Asbestosis and Cancer, People and Work, Research Reports* **14**: 92–96, Finnish Institute of Occupational Health: Helsinki, Finland

Eigenwillig GG (1997) Berufliche Strahlenexposition durch Radon und dessen Folgeprodukte. Konsequenzen für die Anerkennung als Berufskrankheit. *Dtsch Ärzteblatt* **94**: 1057–1062

Fontanini G, Fiore L, Bigini D, Vignati S, Calvo S, Mussi A, Lucci M, Angeletti CA and Merlo Basolo F (1994) Levels of p53 antigen in serum of non-small cell lung cancer patients correlate with positive p53 immunohistochemistry on tumor sections, tumor necrosis and nodal involvment. *Int J Oncol* **5**: 553–558

Haeder M, Rotsch M, Bepler G, Henning C, Havemann K, Heimann B and Moelling K (1988) Epidermal growth factor receptor expression in human lung cancer cell lines. *Cancer Res* **48**: 1132–1136

Hanaoka T, Yamano Y, Katsuno N, Kagawa J and Ishizu S (1997) Elevated serum levels of pantropic p53 in chronium workers. *Scand J Work Environ Health* **23**: 37–40

Harris CC and Hollstein M (1993) Clinical implications of the p53 tumor-suppressor gene. *New Engl J Med* **329**: 1318–1327

Horio Y, Suzuki H, Ueda R, Koshikaea T, Sigura T, Ariyoshi Y, Shimokata K and Takahashi T (1994) Predominantly tumor-limited expression of a mutant allele in a Japanese family carrying a germline p53 mutation. *Oncogene* **9**: 1231–1235

Husgafvel-Pursiainen K, Kannio A, Oksa P, Suitiala T, Koskinen R., Hemminiki K, Smith S, Rosenstock-Leibu R and Brandt-Rauf P (1997) Mutations, tissue accumulations, and serum levels of p53 in patients with occupational cancers from asbestos and silica exposure. *Environ Mol Mutagen* **30**: 224–230

Kaye FJ, Kim YW and Otterson GA (1995) Molecular biology of lung cancer. In: *Molecular Genetics of Cancer*, Cowell JK (ed.), pp. 179–204. BIOS Scientific: Oxford

© 1999 Cancer Research Campaign

Lahdeaho ML, Lehtinen T, Aine R, Hakala T and Lehtinen M (1994) Antibody response to adenovirus E1b-derived synthetic peptides and serum levels of p53 in patients with gastrointestinal and other malignant lymphomas. *J Med Virol* **43**: 393–396

Levesque MA, D'Casta M and Diamandis EP (1996) p53 protein is absent from serum of patients with lung cancer. *Br J Cancer* **74**: 1434–1440

Lubin R, Zalcman G, Bouchet L, Tredaniel J, Lagros Y, Cazals D, Hirsch A and Soussi T (1995) Serum p53 antibodies as early markers of lung cancer. *Nat Med* **1**: 701–702

Luo JC, Zehab R, Antilla S, Ridanpää M, Husgafvel-Pursiainen K, Vaino H, Carney W, DeVivo J, Milling C and Brandt-Rauf PW (1994) Detection of serum p53 protein in lung cancer patients. *J Occup Med* **36**: 155–160

Micelli G, Donaded A and Quaranta M (1992) The p53 tumor suppressor gene. A preliminary clinical study in breast cancer patients. *Cell Biophys* **21**: 25–31

Minna JD (1993) The molecular biology of lung cancer pathogenesis. *Chest* **103**: 449–456

Mudena B, Green JA, Green B, Jenkins JR, Robertson L, Tarunina M and Leinster SJ (1994) The relationship between serum p53 autoantibodies and characteristics of human breast cancer. *Br J Cancer* **69**: 1115–1119

Nuorva U, Makitaro R, Huhti E, Kamel D, Vähäkangas K, Bloigu R, Soini Y and Paakko P (1994) p53 protein accumulation in lung carcinomas of patients exposed to asbestos and tobacco smoke. *Am J Respir Crit Care Med* **150**: 528–533

Parker JE (1997) Radiological criteria: the use of chest imaging techniques in asbestos-related diseases. In: *Proceedings of an International Expert Meeting on Asbestos, Asbestosis and Cancer, People and Work, Research Reports* **14**: 28–40, Finnish Institute of Occupational Health: Helsinki, Finland

Partanen R, Hemminki K, Brandt-Rauf PW, Jin CG and Koskinen H (1994*a*) Serum levels of growth factor receptors, EGFR and Neu in asbestosis patients: A follow-up study. *Int J Oncol* **4**: 1025–1028

Partanen R, Hemminki K, Koskinen H, Luo JC, Carney WP and Brandt-Rauf PW (1994*b*) The detection of increased amounts of the extracellular domain of the epidermal growth factor receptor in serum during carcinogenesis in asbestosis patients. *J Occup Med* **36**: 1324–1328

Pavelic K, Banjac Z, Pavelic J and Spaventi S (1993) Evidence for a role of EGF receptor in the progression of human lung carcinoma. *Anticancer Res* **14**: 1133–1137

Preudhomme C, Lubin R, Lepelley P, Vanzumbeke M and Fenaux P (1994) Detection of serum anti p53 antibodies and their correlation with p53 mutations in myelodysplastic syndrom and acute myeloid leukemia. *Leukemia* **8**: 1589–1591

Rosenfeld MR, Malats N, Schramm L, Graus F, Cardenal F, Vinolas N, Rosell R, Tora M, Real FX, Posner JB and Dalmau J (1997) Serum anti-p53 antibodies and prognosis of patients with small-cell lung cancer. *J Natl Cancer Inst* **89**: 381–385

Rusch V, Baselga J, Cordon-Cardo J, Orazem J, Zaman M, Hoda S, McIntosh J, Kurie J and Dmitrovsky E (1993) Differential expression of the epidermal growth factor receptor and its ligands in primary non-small-cell lung cancers and adjacent benign lung. *Cancer Res* **53**: 2379–2385

Schlichtholz B, Tredaniel J, Lubin R, Zalcman G, Hirsch A and Soussi T (1994) Analysis of p53 antibodies in sera of patients with lung carcinoma define immunodominant regions in the p53 protein. *Br J Cancer* **69**: 809–816

Segawa Y, Tagigawa N, Mandai K, Maeda Y, Takata I, Fujimoto N and Jnno K (1997) Measurement of serum p53 protein in patients with small cell lung cancer and results of its clinicopathological evaluation. *Lung Cancer* **16**: 229–238

Sorscher SM, Russak V, Graziano V, Cagle M, Feramisco JR and Green MR (1995) Immunohistochemical evaluation of E-cadherin and epidermal growth factor receptor in non-small-cell lung cancer. *Mod Pathol* **8**: 450–455

Sozzi G, Miozzo M, Tagliabue E, Calderone C, Lombardi L, Pilotti S, Pastorino U, Pierotti MA and DellaPorta G (1991) Catogenetic abnormalities and overexpression of receptors for growth factors in normal bronchial epithelium and tumor samples of lung cancer patients. *Cancer Res* **51**: 400–404

Sugimura H, Weston A, Caporaso N, Shields PG, Bowman ED, Metcalf RA and Harris CC (1991) Biochemical and molecular epidemiology of cancer. *Biomed Environ Sci* **4**: 73–92

Taylor JA, Watson MA, Devereux TR, Michels RY, Saccomanno G and Anderson M (1994) p53 mutations hotspot in radon-associated lung cancer. *Lancet* **343**: 86–87

Trivers GE, Cawley HL, DeBenedetti VM, Hollstein M, Marion MJ, Bennett WP, Hoover ML, Price CC, Tamburro CC and Harris CC (1995) Anti-p53 antibodies in sera of workers occupationally exposed to vinyl chloride. *J Natl Cancer Inst* **87**: 1400–1407

Trumper L, Jung W, Dahl G, Diehl V, Gause A and Pfrundschuh M (1994) Interleukin-7, interleukin-8, soluble TNF receptor and p53 protein levels are elevated in the serum of patients with Hodgkin's disease. *Ann Oncol* **5**: 93–96

Vähäkangas KH, Samet JM, Metcalf RA, Welsh JA, Bennett WP, Lane DP and Harris CC (1992) Mutations of p53 and ras genes in radon-associated lung cancer from uranium miners. *Lancet* **339**: 576–580

van Zandwijk N, Mooi WJ and Rodenhuis S (1995) Prognostic factors in NSLCL. Recent experiences. *Lung Cancer* **12**: 27–33

Weissfeld JL, Larsen RD, Niman HL and Kuller LH (1994) Evaluation of oncogene-related proteins in serum. *Cancer Epidemiol Biomarkers Prev* **3**: 57–62

Wild CP, Ridanpää M, Antilla S, Lubin R, Soussi T, Husgavfel-Pursianen K and Vainio H (1995) p53 antibodies in the sera of lung cancer patients: comparison with p53 mutations in the tumor tissue. *Int J Cancer* **64**: 176–181

Winter SF, Minna JD, Johnson BE, Takahashi T, Gazdar AF and Carbone DP (1992) Development of antibodies against p53 in lung cancer patients appears to be dependent on the type of p53 mutation. *Cancer Res* **52**: 4168–4174

Winter SF, Sekido Y, Minna JD, McIntire D, Johnson BE, Gazdar AF and Carbone DP (1993) Antibodies against autologous tumour cell proteins in patients with small-cell lung cancer: associated with improved survival. *J Natl Cancer Inst* **85**: 2018

Wynford-Thomas D (1991) Oncogenes and anti-oncogenes; the molecular basis of tumour behaviour. *J Pathol* **165**: 187–201

*© 1999 Cancer Research Campaign*
